# Pulmonary nodules in a patient with rheumatoid arthritis: Which diagnostic approach is the most appropriate?

**DOI:** 10.22088/cjim.8.3.220

**Published:** 2017

**Authors:** Ines Mahmoud, Aicha Ben Tekaya, Rawdha Tekaya, Olfa Saidane, Leila Gafsi, Mathilde Benhammou, Fautrel Bruno, Leila Abdelmoula

**Affiliations:** 1 Department of Rheumatology, Charles Nicolle, Tunis, Tunisia.; 2 Department of Rheumatology, Pitié Salpetrière Hospital, Paris, France.

**Keywords:** Pulmonary nodule, Rheumatoid arthritis, Epidermoid carcinoma, Tumor cavitation, Computed tomography

## Abstract

**Background:**

Pulmonary nodular excavation should firstly evoke tuberculosis or necrosis broncho-pulmonary tumor, particularly: epidermoid carcinoma. The case discussed here illustrated these difficulties in patients with rheumatoid arthritis (RA).

**Case Presentation:**

A 63-year-old woman was presented with a-three-year history of RA and a recent discovery of an excavated pulmonary nodule. Initial investigations focused on a rheumatoid origin. The evolution of the disease was worrisome and surgical exploration was deemed mandatory. The result was the discovery of a nodule of a malignant nature.

**Conclusion:**

In this paper, we discussed the excavation of the pulmonary nodule, its diagnoses and management of the difficulties we encountered.

**R**arely, epidermoid carcinoma can have a nodular appearance in x-ray imaging. In 10% of cases, the excavation shows thickened walls and a nodular internal surface (1). Yet, pulmonary nodular excavation should firstly evoke tuberculosis or necrosis broncho-pulmonary tumor, particularly: epidermoid carcinoma (2). Some diagnostic difficulties may arise despite the current sophisticated means of investigations. The case discussed here illustrated these difficulties in patients with RA. 

## Case Presentation

**Figure 1:**
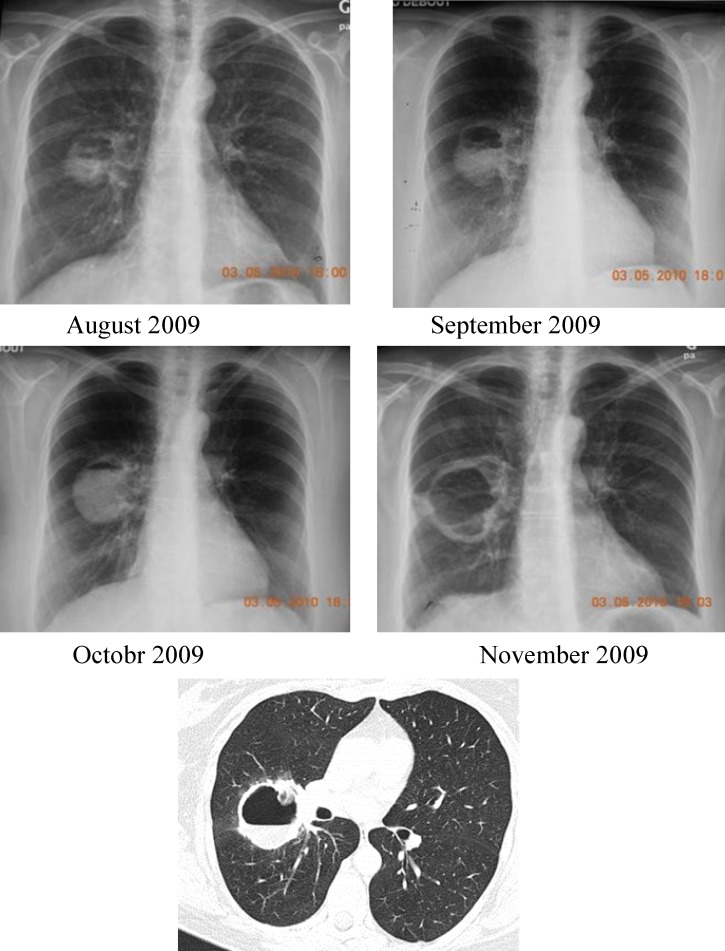
A monthly radiological re-evaluation revealing a gradual increase in size of the nodule and since September, a progressive emergence of an excavation with a variable fluid level according to x-rays. The CT scan confirms this aspect

A 63-year-old woman was presented with a-three-year history of rheumatoid arthritis (RA) and a recent discovery of a pulmonary nodule. Her RA is erosive, sero-positive (RF and anti-CCP) and was never treated with a disease modifying anti-rheumatic drug **(**DMARD**)**. Moreover, she smoked for 30 years and stopped 12 years ago. As part of the pre-therapeutic checkup of the RA, chest radiography was done and showed a suspect nodule. Chest computed tomography was then performed in July, 2009. This CT revealed a pulmonary nodule measuring 3 cm across the 3 lobes of the right lung and an axillary adenopathy with no suspect aspect of malignancy. Several diagnoses were considered including: infectious origin, cancer, rheumatoid nodule and so on. A PET scan was then performed. A hypermetabolism of the pulmonary nodule (SUV max to 4.2) associated with hypermetabolism of two contralateral axillary adenopathies (SUV:1.7), having no suspect aspect of malignancy, were found (figure1). The bronchoscopy did not show any abnormalities. Abdominal CT was normal. A biopsy was conducted in August 2009 and was negative. Usual causes of infections were eliminated. However, a therapeutic test with probabilistic broad spectrum antibiotherapy with cotrimoxazole was started for 3 weeks, with no significant impact on the nodule. 

Since infection seemed to be ruled out, a surgical biopsy was discussed. The nodule being present at the intersection of the 3 lobes, the nodule resection meant a right pneumonectomy. The procedure was considered too aggressive with regard to the probability of the nodule being malignant rather than rheumatoid. Thus, after a multidisciplinary discussion, methotrexate was started while doing a monthly radiological surveillance of the nodule. 

In November 2009, a hemoptysis appeared. The monthly radiological re-evaluation revealed a gradual increase in size of the nodule and since September there was a progressive emergence of an excavation with a variable fluid level according to x-rays. CT scan confirms this aspect (figure 1). 

Finally, in January 2010, because of the persistence of the hemoptoic sputum and despite an arterio-embolization, a surgical exploration with a right pneumonectomy was conducted. The data gathered from the procedure concluded on the malignant character of the nodule with a histological appearance of an epidermoid carcinoma. 

The discovery of a pulmonary nodule in RA in the absence of histological signs of malignancy in the transthoracic biopsy, led us initially to a rheumatoid arthritis origin. However, the rapidly evolving nature, the size of the tumor, the occurrence of hemoptysis and hypermetabolic character in the PET scan are arguments enough to support the hypothesis of the malignancy of the nodule. Nonetheless, it remains difficult to differentiate a rheumatoid nodule from a bronchial carcinoma, in particular, in a smoker patient. In the case of a single nodule, hypermetabolism can be observed during a PET scan using fluorodeoxyglucose as biomarker (3). The rheumatoid nodule can be large and as well, complicated with an excavation, a hemorrhagia, an infection or bronchopleural fistula. In this case, a histological confirmation is required (4). 

An iconographic review of 50 radiological records with localized pulmonary nodular lesions (or masses) excavated, helped identifying diagnostic clues to mention. Causes of excavated nodules were dominated by malignant tumors and community infections (5) in this series. 

During the 2007 ATS (American Thoracic Society), several mathematical models were discussed to establish the likelihood of an isolated nodule malignancy. The reference model is that of Swensen [6], said of the Mayo Clinic, dating back to 1997. The pre-test, probability of malignancy takes into account three independent clinical variables (age, cigarette-smoking status, history of extra thoracic cancer) and three independent radiological variables (diameter, spiculation, localization in the upper lobe). Gould et al. (7) have established four independent predictive factors of malignancy: cigarette-smoking status (OR: 7.9; 95% CI: 2.6-23.6), age (OR: 2.2; 95% CI: 1.7-2.8), size of the nodule (OR: 1.1; 95% CI: 1.1-1.2) and duration of the cessation (OR: 0.6; 95% CI: 0.5-0.7). The probability of malignancy is higher in older former smoker patients having not stopped smoking with large nodules. 

 Herder et al. (8) assessed added value of the PET scan to the predictive model of Swensen in a population of 106 patients among whom had malignant nodules. The Swensen template has confirmed its reliability. The contribution of the PET scan is of interest in the indeterminate and classified low risk of malignancy nodules. Gould et al. (9) have also established recommendations and clinical practice guidelines for detecting lung cancer. If we consider the validated model of Mayo Clinic, our patient is of intermediate risk of cancer with 57% probability of malignancy. Considering the following recommendations: the fact that the nodule is hypermetabolic by FDG-PET imaging (15th recommendation) and the clear evidence of growth of the nodule on imaging tests (second recommendation), tissue diagnosis should be obtained.

In conclusion our observation underlines that sometimes the misleading nature of the pulmonary nodule observed in the RA, as well as the technical difficulties support the diagnosis. Because of the fear of the possibility of having cancer, diagnostic approaches discouraged people to move farther. 

## Conflicts of Interest:

There is no conflict of interest
